# The Non-Residential Preliminary School

**Published:** 1921-07-23

**Authors:** 


### THE MATRONS' AND SISTERS' DEPARTMENT.

### THE NON-RESIDENTIAL PRELIMINARY SCHOOL.

It is pretty generally recognised that observance of the Draft Syllabus of Training put forth by the General Nursing Council must involve a period of preliminary instruction given to probationers before they enter on their duties in the wards. One institution after another is adding such a school for candidates to its ordinary training-school, and some, like the Royal Halifax Infirmary, as noted last week, are enlarging their regular course from three to four years.

In the present heavily burdened state of voluntary hospitals, not to mention the wretched plight of the ratepayers, on whose earnings the Poor-law institutions depend, the proposal to house an additional group of probationers comes as an unwelcome surprise to managers. In many hospitals it is already impossible to find accommodation for the nurses required to bring working conditions into line with modern requirements. If, over and above the necessary staff, a demand is made for providing a suite of rooms in which candidates can enjoy the comforts and teaching facilities of a residential college without performing any services in the wards, the prospect from a financial point of view becomes very dark. No doubt some part of the cost can be recovered from the pupil nurse, though even that is doubtful in view of the poverty of some of the best candidates. But to conceive that such a residential college could ever pay its way and be entirely self-supporting as to rent, rates, salaries, tuition, and general upkeep seems to us far too optimistic. In institutions so happy as to contain surplus accommodation or to have had a building presented for this purpose, it may be possible to make the preliminary school pay. The rest must, we believe, be content to give a non-residential course to women who desire to become probationers.

It by no means follows that each hospital must arrange a special course for its own candidates. The time has come when courses suited to prepare women for the duties of probationers could with advantage be arranged by public bodies, and we commend to our readers Miss Sheldon's letter on this subject, which appeared in last week's issue, page 268. The Draft Syllabus gives the lead which has long been wanting in this respect. The lessons, which probationers in the first rush of assimilating impressions of hospital life are expected painfully to master, when too wearied with unaccustomed routine to use their brains clearly, can and ought to be conned long before the threshold of the institution has been crossed. The elements of anatomy and physiology should be familiar to the student long before the suggestion of diseased conditions in patients under care intervenes to cloud her vision. Bed-making, the cleansing of instruments and utensils, the treatment of floors, and other practical details should be practised until the student is ready to be a help instead of a nuisance in a busy ward. Cookery, not merely from the fanciful invalid's point of view, but the essential principles of preparing food, should be mastered by the woman who, entering the nursing service, is fitting herself to have charge of other people's welfare. Note-taking, simple accounts, and business routine should certainly form a part of the nurse's preparation for training. And a six months' course would not prove too long.

The great advantage of such a preliminary course, presided over by a nursing sister of proved experience and powers of teaching, would be that it would draw in a great number of promising candidates who shrink from plunging into hospital life without some such preparation, and who could usefully employ their time in this way between the period of leaving school and adopting a career. The entrance age need not be over eighteen, and the fees for five days' tuition a week need not, under non-residential conditions, make great demands on the purse. The leaving examination, coupled with the report of the sister-in-charge, would test the candidates, and there is no reason to doubt that sufficient applications from training-schools would be received to find places for every one who had acquitted herself well.

It may be objected that the main value of a period of residential training lies in its power to train the character. But the probationer will still have three or four years in which to be moulded, and it is a disputed point whether she gains or loses by too protracted a term of residence in an institution. If the hospital cannot make her what it wants during her training, it is certain the preliminary school will not have any permanent effect.

